# Metabolic evaluation of urolithiasis: a narrative review

**DOI:** 10.1007/s00120-025-02617-2

**Published:** 2025-06-20

**Authors:** Panagiotis Angelopoulos, Panagiotis Neofytoy, Stamatios Katsimperis, Panagiotis Triantafylloy, Ioannis Manolitsis, Michael Lardas, Titos Markopoulos, Ioannis Varkarakis, Lazaros Tzelves, Andreas Skolarikos

**Affiliations:** Second Department of Urology, National and Kapodistrian University of Athens, Sismanogleio General Hospital, Athens, Greece

**Keywords:** Urolithiasis, Metabolic evaluation, Follow-up, Kidney stones, Endourology, Urolithiasis, Metabolische Untersuchung, Follow-up, Nierensteine, Endourologie

## Abstract

Urolithiasis is a common condition with high recurrence rates, often driven by metabolic abnormalities, dietary habits, and systemic disorders. Metabolic evaluation plays a key role in identifying modifiable risk factors such as hypercalciuria, hyperoxaluria, hypocitraturia, and hyperuricosuria. Despite its clinical value, metabolic evaluation remains underused in practice. This narrative review highlights the rationale, methods, and therapeutic implications of metabolic assessment in patients with recurrent or high-risk stone disease. The evaluation includes medical and dietary history, physical examination, blood and urine tests, 24‑h urine collections, and stone analysis. Tailored interventions—ranging from dietary advice to pharmacologic treatments such as thiazides, potassium citrate, and allopurinol—can reduce stone recurrence and prevent complications such as chronic kidney disease. Ongoing follow-up is essential to assess treatment response and modify strategies. Adherence to current guidelines and broader implementation of metabolic workup can significantly improve patient outcomes and reduce the global burden of stone disease.

## Abstract (de)

Urolithiasis ist eine häufige Erkrankung mit hohen Rezidivraten, die oft durch metabolische Anomalien, Ernährungsgewohnheiten und systemische Erkrankungen begünstigt wird. Die Stoffwechseluntersuchung spielt eine entscheidende Rolle bei der Erkennung beeinflussbarer Risikofaktoren wie Hyperkalziurie, Hyperoxalurie, Hypozitraturie und Hyperurikosurie. Trotz ihres klinischen Nutzens wird die Untersuchung des Stoffwechsels bisher zu wenig in der Praxis eingesetzt. In der vorliegenden narrativen Übersichtsarbeit werden die Gründe, Methoden und therapeutischen Auswirkungen der Stoffwechseluntersuchung bei Patienten mit rezidivierendem oder hohem Risiko für Steinleiden dargestellt. Dazu gehören die Anamnese in Bezug auf die medizinische Vorgeschichte und Ernährungsgewohnheiten, die körperliche Untersuchung, Blut- und Urinuntersuchungen, die 24-h-Urinsammlung und die Steinanalyse. Maßgeschneiderte Interventionen – von Ernährungsberatung bis zur pharmakologischen Behandlung, z. B. mit Thiaziden, Kaliumzitrat und Allopurinol, – können Rezidive der Steinbildung vermindern und Komplikationen wie der chronischen Niereninsuffizienz vorbeugen. Eine fortlaufende Nachuntersuchung ist essenziell für die Beurteilung des Therapieansprechens und Modifikationen der Behandlungsstrategie. Durch Befolgen der aktuellen Leitlinien und die breitere Implementierung der Untersuchung des Stoffwechsels können die Ergebnisse für die Patienten deutlich verbessert und die globale Belastung durch Steinerkrankungen vermindert werden.

## Introduction

Urolithiasis is a common disorder with increasing global incidence, largely attributed to lifestyle changes, dietary habits, and metabolic predispositions [1–4]. The risk of recurrence is high, with approximately 50% of patients developing a new stone within 5 years and up to 80% within a decade [5]. Understanding the metabolic basis of stone formation is essential to implementing targeted preventive measures. Despite its importance, the application of metabolic evaluation varies among urologists worldwide, as highlighted in some previous reports, indicating that metabolic evaluation is underutilized in clinical practice [6–8].

Metabolic evaluation aims to identify modifiable risk factors contributing to stone formation, such as hypercalciuria, hyperoxaluria, hypocitraturia, and metabolic acidosis. Identifying these factors allows for tailored dietary and pharmacologic interventions, reducing recurrence rates and preventing complications such as chronic kidney disease. While first-time stone formers can benefit from general dietary advice, recurrent stone formers and those with high-risk profiles require comprehensive metabolic assessment to identify underlying metabolic disorders, including renal tubular acidosis, primary hyperparathyroidism, and enteric hyperoxaluria.

## Epidemiology and risk factors

Urolithiasis has seen a dramatic increase in prevalence over the past few decades, with variations in incidence based on geographic location, dietary habits, and genetic predisposition. Studies indicate that the prevalence of kidney stones ranges from 5% to 15% in developed nations, with higher rates in regions with warmer climates and lower water intake [3]. The increasing incidence is correlated with rising rates of obesity, metabolic syndrome, diabetes, and dietary shifts toward high-sodium, high-protein, and low-fiber diets [3, 4].

Risk factors for urolithiasis include:

- *Dietary factors*: High intake of sodium, oxalate, animal proteins, and refined sugars has been linked to increased stone formation. Conversely, diets rich in citrate (e.g., citrus fruits) and adequate calcium intake can be protective.
- *Metabolic conditions*: Hypercalciuria, hyperoxaluria, hypocitraturia, and hyperuricosuria significantly contribute to stone formation. Systemic conditions such as hyperparathyroidism, renal tubular acidosis, and metabolic syndrome exacerbate stone risk.
- *Genetic predisposition*: Family history is a strong predictor of stone disease, with monogenic disorders such as cystinuria and primary hyperoxaluria leading to recurrent and severe stone formation.
- *Environmental and lifestyle factors*: Dehydration, sedentary lifestyle, and chronic low urine output contribute to urinary supersaturation, a critical factor in stone formation.

## Rationale for metabolic evaluation

Metabolic evaluation is a crucial component of managing recurrent urolithiasis, yet its utilization remains inconsistent. Many urologists focus on surgical management of stones without adequately addressing underlying metabolic abnormalities, leading to high recurrence rates. The primary goals of metabolic evaluation include:

1. Identifying modifiable dietary and lifestyle risk factors
2. Detecting systemic diseases contributing to stone formation
3. Classifying patients based on recurrence risk and guiding individualized treatment
4. Preventing stone recurrence and reducing long-term morbidity, including chronic kidney disease

The European Association of Urology (EAU) and American Urological Association (AUA) guidelines emphasize the need for a thorough metabolic workup, particularly in high-risk patients [9, 10]. However, studies indicate that metabolic evaluation is underperformed, with only 7–16% of stone formers undergoing comprehensive 24‑h urine testing [6–8].

## Diagnostic approach to metabolic evaluation

A systematic and structured diagnostic approach is essential for an effective metabolic evaluation in patients with nephrolithiasis. The goal of this evaluation is to identify underlying metabolic abnormalities, assess risk factors, and develop personalized preventive strategies to reduce stone recurrence. The key components of the diagnostic process include medical and dietary history, physical examination, laboratory investigations, imaging studies, and stone analysis.

### Medical and dietary history

A comprehensive medical and dietary history serves as the cornerstone of metabolic evaluation, providing essential insights into potential risk factors for stone formation.

A thorough personal and family history should be obtained, including details about previous kidney stone episodes, frequency of recurrence, and prior interventions such as surgical procedures or medical therapies. A positive family history of nephrolithiasis may indicate a genetic predisposition to stone formation, highlighting the need for closer metabolic monitoring.

Dietary habits play a significant role in the pathogenesis of kidney stones, and a detailed dietary assessment should be conducted. Particular attention should be given to calcium intake, as both excessive and inadequate consumption can influence stone risk. High dietary sodium promotes urinary calcium excretion, increasing the likelihood of calcium-based stones, while excessive animal protein intake contributes to acid load, hypercalciuria, and hypocitraturia [11]. Oxalate-rich foods, such as spinach, nuts, and chocolate, may elevate urinary oxalate levels, increasing the risk of calcium oxalate stone formation [12]. Inadequate fluid intake is a well-recognized risk factor for nephrolithiasis, leading to low urinary volume and supersaturation of stone-forming solutes [13].

The patient’s medication use should also be carefully reviewed, as certain drugs can influence stone risk. Thiazide diuretics reduce urinary calcium excretion and may be beneficial in patients with recurrent calcium stones, while loop diuretics increase calcium excretion and may promote stone formation. Calcium supplements, particularly when taken without food, can contribute to hypercalciuria [14]. Other medications, such as protease inhibitors and excessive vitamin C supplementation, have also been associated with nephrolithiasis [15].

Lastly, an assessment of systemic conditions associated with kidney stone formation is critical. Hyperparathyroidism is a major contributor to hypercalcemia and hypercalciuria, leading to recurrent calcium-based stones [16]. Inflammatory bowel disease and other malabsorption syndromes can increase intestinal oxalate absorption, resulting in enteric hyperoxaluria and subsequent calcium oxalate stone formation. Patients with gout and hyperuricemia are at increased risk for uric acid stones due to persistently acidic urine, while metabolic syndrome and diabetes mellitus are associated with aciduria, increased uric acid excretion, and heightened susceptibility to uric acid nephrolithiasis [17].

### Physical examination

A focused physical examination provides additional insights into metabolic and systemic risk factors for stone formation. Measurement of body mass index (BMI) and blood pressure is essential, as obesity and hypertension are independently associated with an increased risk of nephrolithiasis [18, 19]. In patients with suspected hyperparathyroidism, signs of skeletal abnormalities such as bone pain or fractures may be present due to increased bone turnover. Signs of dehydration, including reduced skin turgor and dry mucous membranes, should also be evaluated, as inadequate hydration is a major contributor to stone formation.

### Laboratory testing

Laboratory investigations play a crucial role in identifying metabolic abnormalities that contribute to nephrolithiasis.

Blood analysis should always be a part of the metabolic workup including at least creatinine, uric acid, (ionized) calcium, sodium, potassium, blood cell count, and C‑reactive protein. Measurement of total and ionized calcium levels, along with parathyroid hormone (PTH), helps diagnose hyperparathyroidism in patients with hypercalcemia. Serum phosphorus levels provide additional insights into calcium–phosphorus balance, which is important for parathyroid function assessment. Elevated serum uric acid levels may indicate an increased risk of uric acid stone formation, while serum bicarbonate measurements help detect metabolic acidosis, a condition that contributes to hypocitraturia and calcium phosphate stone formation. In patients with suspected metabolic syndrome, blood glucose and insulin levels should be assessed, as insulin resistance is strongly linked to low urinary pH and uric acid nephrolithiasis.

Urinalysis provides valuable information about urinary pH, specific gravity, and the presence of crystalluria, which may suggest the type of stone being formed. A urine culture is necessary to rule out infection-related stones, such as struvite stones associated with urease-producing bacteria. It is recommended to perform 24‑h urine collection for high-risk patients, particularly those with recurrent or complex nephrolithiasis. Specific metabolic evaluation requires the collection of two consecutive 24‑h urine samples although there are data indicating that even one 24‑h urine sample might be adequate [20]. The collecting bottles should be prepared with 1 g/L thymol or stored at < 8 °C during collection to reduce bacterial proliferation [21]. This test provides a comprehensive assessment of urinary excretion of key solutes, including calcium, oxalate, citrate, uric acid, sodium, magnesium, and creatinine. Elevated urinary calcium suggests hypercalciuria, a major risk factor for calcium-containing stones. Increased urinary oxalate levels may indicate excessive dietary intake or underlying gastrointestinal disorders. Low urinary citrate is a key risk factor for calcium stone formation, as citrate acts as a natural inhibitor of crystal aggregation. High urinary uric acid levels increase the likelihood of uric acid stone formation, particularly in acidic urine. Measurement of urinary sodium helps evaluate dietary salt intake, which influences urinary calcium excretion. Magnesium levels are also assessed, as magnesium plays a protective role by inhibiting calcium crystallization. Creatinine excretion is measured to confirm the adequacy of urine collection.

Stone analysis is essential for guiding further metabolic evaluation and treatment strategies in all first-time stone formers according to the European Association of Urology (EAU) guidelines. By contrast, the American Urological Association (AUA) guidelines state that a stone analysis should be performed at least once when a stone is available, because it may help direct preventive measures. The preferred techniques are infrared spectroscopy or X‑ray diffraction [22]. They are used to determine stone composition, which helps in identifying the specific metabolic derangements contributing to stone formation.

For the initial metabolic evaluation, patients should maintain their usual diet and lifestyle, and it is preferable that they remain stone-free for a minimum of 20 days. Ongoing monitoring is recommended for individuals receiving medications aimed at preventing recurrence. The first 24‑h urine collection should be carried out 8–12 weeks after initiating pharmacological therapy. Once urinary parameters are stabilized, annual 24‑h urine assessments are generally adequate. It should be noted, however, that the supporting evidence in the literature for these recommendations is limited.

## Risk stratification and interpretation of results

The metabolic evaluation of patients with nephrolithiasis not only helps identify underlying risk factors but also aids in stratifying patients based on their risk profile. This classification guides the intensity of metabolic assessment and the need for targeted preventive strategies.

Low-risk patients include first-time stone formers with no identifiable metabolic disorders and no significant risk factors for recurrence. These individuals are primarily managed with lifestyle modifications aimed at reducing stone formation risk. Given the absence of strong predisposing factors, detailed metabolic evaluation is typically not required unless there is a subsequent recurrence.

By contrast, high-risk patients include those with recurrent stone formation, early-onset nephrolithiasis (especially in childhood or adolescence), a strong familial history of kidney stones, underlying metabolic disorders, or a high stone burden. Patients with conditions such as hyperparathyroidism, inflammatory bowel disease, renal tubular acidosis, or cystinuria fall into this category. These individuals require a more comprehensive metabolic workup, including detailed laboratory testing and 24‑h urine analysis, to identify specific abnormalities contributing to stone formation. The goal in this group is to implement individualized preventive strategies to reduce recurrence and associated complications.

## Therapeutic implications of metabolic evaluation

The findings from the metabolic evaluation provide a foundation for the implementation of personalized preventive and therapeutic interventions. These strategies include dietary modifications, pharmacologic therapy, and long-term follow-up to minimize stone recurrence.

### Dietary modifications

Dietary interventions form the cornerstone of kidney stone prevention and are tailored to the patient’s specific metabolic profile. A fundamental recommendation for all stone formers is to increase fluid intake, ensuring sufficient urine output to dilute stone-forming solutes and reduce urinary supersaturation [23]. A fluid intake of 2.5–3 L/day is considered to be adequate. The beneficial effect of fruit juices is mainly determined by the presence of citrate or bicarbonate. Citrus fruit juices seem to protect against stone disease either by increasing urinary citrate levels or by having an alkalinizing effect on it [24].

Calcium intake should be maintained within the normal recommended range (1–1.2 g/day) to prevent increased intestinal oxalate absorption, which can contribute to calcium oxalate stone formation. Contrary to previous misconceptions, calcium restriction is generally discouraged, as inadequate dietary calcium leads to enhanced oxalate absorption and increased stone risk. Calcium supplements are not recommended except in enteric hyperoxaluria when additional calcium should be taken with meals to bind intestinal oxalate.

Reducing sodium intake is particularly important for patients with hypercalciuria, as high sodium intake increases urinary calcium excretion due to competition for renal tubular reabsorption. Generally, daily sodium intake should not exceed 4–5 g. Patients should be advised to limit processed foods and avoid excessive salt consumption. Despite these considerations, there are no data from prospective trials demonstrating that sodium restriction is associated with a reduced risk of stone formation.

For patients with hypocitraturia, increasing dietary citrate intake through the consumption of citrus fruits (e.g., lemons, oranges) is recommended. Citrate acts as a natural inhibitor of calcium oxalate crystallization by binding calcium in the urine, thereby preventing stone formation.

For patients with hyperoxaluria, dietary restriction of oxalate-rich foods such as spinach, nuts, tea, and chocolate is advised. However, rather than complete avoidance, a balanced approach that includes sufficient dietary calcium intake to bind oxalate in the gut is preferred.

### Pharmacologic interventions

In cases where dietary modifications alone are insufficient to correct metabolic abnormalities, pharmacologic therapy is required to optimize urinary chemistry and prevent stone recurrence.

Thiazide diuretics (e.g., hydrochlorothiazide, chlorthalidone) are prescribed for patients with hypercalciuria, as they reduce urinary calcium excretion by promoting calcium reabsorption in the renal tubules. These medications are particularly beneficial in recurrent calcium stone formers. However, their value has recently been questioned. A recent randomized controlled trial concluded that thiazide does not differ significantly from placebo in preventing kidney stone recurrence in high-risk patients [25]. Notably, an examination of the study protocol reveals that it was powered to detect the existence of a dose–response relationship, and the study results showed a linear trend for the effect of three different hydrochlorothiazide doses (12.5, 25, and 50 mg/day) on stone recurrence [26]. Therefore, the study’s conclusion should be interpreted with caution.

Potassium citrate supplementation is used for hypocitraturia, a common metabolic abnormality in recurrent calcium oxalate and uric acid stone formers. By increasing urinary citrate levels, potassium citrate helps prevent crystal aggregation and reduces stone formation in hypocitraturic as well as hyperuricosuric stones formers.

Allopurinol is indicated for patients with hyperuricosuria as a first-line treatment. By reducing uric acid production, allopurinol lowers urinary uric acid excretion and helps prevent stone recurrence. Another indication is in patients with 2,8-dihydroxyadenine stones. High-dose allopurinol and febuxostat are important options but should be given with regular monitoring [27].

Sodium bicarbonate or potassium bicarbonate therapy is beneficial for patients with metabolic acidosis-related stones, such as those with renal tubular acidosis. The dose for those with renal tubular acidosis is 1.5 g three times daily. By alkalinizing the urine, these agents prevent uric acid and cystine stone formation while promoting calcium solubility. The pH should be adjusted to 7.0–7.2. The EAU guideline recommendation for sodium bicarbonate in cases of hypocitraturia carries a strong strength rating, underlining their importance as an efficacious pharmacologic option.

### Long-term follow-up

Ongoing monitoring and follow-up are essential to ensure the effectiveness of preventive measures and assess patient adherence. Regular metabolic reassessment allows for adjustments in dietary and pharmacologic interventions based on urinary parameters.

Follow-up after definitive treatment for upper urinary tract stones remains an area of limited consensus due to the heterogeneity of stone disease and the paucity of comparative studies. The EAU Urolithiasis Guidelines Panel performed a systematic review that helped them reach a consensus on the ideal follow-up schedule [28]. For stone-free patients, follow-up can be concluded after 2 years for radiopaque stones or 3 years for radiolucent stones, given that 80% remain stone-free thereafter. For a higher safety margin (90%), surveillance may extend to 5 years. Patients with metabolic abnormalities not on medical treatment show a significantly higher recurrence risk, warranting more intensive monitoring. Periodic imaging studies should include kidney–ureter–bladder X‑ray and/or ultrasound, while computed tomography should be reserved for symptomatic cases or preoperative planning to minimize radiation exposure.

## Conclusion

Metabolic evaluation is a cornerstone in the management of recurrent urolithiasis. While its implementation varies globally, adherence to guideline-based assessments can significantly reduce recurrence rates and improve patient outcomes. Future research should focus on refining risk prediction models and integrating genetic markers into metabolic evaluation protocols. Additionally, increasing awareness and education among urologists on the importance of metabolic evaluation is critical in optimizing stone prevention strategies and reducing the overall burden of urolithiasis.

## Declarations

This article does not contain any studies with human participants or animals performed by any of the authors.
